# Evaluation of Specific Absorption Rate as a Dosimetric Quantity for Electromagnetic Fields Bioeffects

**DOI:** 10.1371/journal.pone.0062663

**Published:** 2013-06-04

**Authors:** Dimitris J. Panagopoulos, Olle Johansson, George L. Carlo

**Affiliations:** 1 Department of Biology, University of Athens, Athens, Greece; 2 Radiation and Environmental Biophysics Research Centre, Athens, Greece; 3 Experimental Dermatology Unit, Department of Neuroscience, Karolinska Institute, Stockholm, Sweden; 4 The Science and Public Policy Institute, Institute for Healthful Adaptation, Washington, D.C., United States of America; Dresden University of Technology, Germany

## Abstract

**Purpose:**

To evaluate *SAR* as a dosimetric quantity for EMF bioeffects, and identify ways for increasing the precision in EMF dosimetry and bioactivity assessment.

**Methods:**

We discuss the interaction of man-made electromagnetic waves with biological matter and calculate the energy transferred to a single free ion within a cell. We analyze the physics and biology of *SAR* and evaluate the methods of its estimation. We discuss the experimentally observed non-linearity between electromagnetic exposure and biological effect.

**Results:**

We find that: a) The energy absorbed by living matter during exposure to environmentally accounted EMFs is normally well below the thermal level. b) All existing methods for *SAR* estimation, especially those based upon tissue conductivity and internal electric field, have serious deficiencies. c) The only method to estimate *SAR* without large error is by measuring temperature increases within biological tissue, which normally are negligible for environmental EMF intensities, and thus cannot be measured.

**Conclusions:**

*SAR* actually refers to thermal effects, while the vast majority of the recorded biological effects from man-made non-ionizing environmental radiation are non-thermal. Even if *SAR* could be accurately estimated for a whole tissue, organ, or body, the biological/health effect is determined by tiny amounts of energy/power absorbed by specific biomolecules, which cannot be calculated. Moreover, it depends upon field parameters not taken into account in *SAR* calculation. Thus, *SAR* should not be used as the primary dosimetric quantity, but used only as a complementary measure, always reporting the estimating method and the corresponding error. Radiation/field intensity along with additional physical parameters (such as frequency, modulation etc) which can be directly and in any case more accurately measured on the surface of biological tissues, should constitute the primary measure for EMF exposures, in spite of similar uncertainty to predict the biological effect due to non-linearity.

## Introduction

Specific Absorption Rate (*SAR*) is defined as the amount of absorbed non-ionizing radiation power (or rate of absorbed energy) by unit mass of biological tissue.

The reason for the introduction of SAR as a non-ionizing radiation – Radio Frequency (RF) Electromagnetic Field (EMF) dosimetric quantity, was – as with the rate of absorbed dose in the ionizing case – to describe the amount of absorbed energy and the rate by which it is absorbed within an exposed tissue and not just the radiation/field intensity on its surface. This derives from the fact that when radiation exposes matter, most usually, it does not interact completely with it and in such a case only a part of its energy gets absorbed. The remainder just passes through without affecting the medium.

The amount of absorbed energy by a certain amount of matter (within a certain time interval) will determine the degree of interaction. But in the case of biological matter this is not as simple. Biological tissue is a much more complicated and organized form of matter compared to inanimate. The degree of interaction does not necessarily determine the biological effect because that depends on which specific bio-molecule – or set of bio-molecules – from a whole tissue or organ will interact with the radiation. Some bio-molecules may get damaged while others may not by the same amount of radiation energy absorbed within the same time-interval.

### Interaction between man-made electromagnetic radiation and living matter

Man-made electromagnetic waves are produced by electromagnetic oscillation circuits (“Thomson” circuits), not by atomic events (as in the case of natural electromagnetic radiation – infrared, visible, ultraviolet, x-rays, γ), and for this they are polarized in contrast to natural electromagnetic radiation that is not. The plane of polarization is determined by the geometry of the circuit. Polarized electromagnetic waves (in contrast to non-polarized) can produce interference effects and induce coherent forced-vibrations on charged/polar molecules within a medium.

When a polarized, non-ionizing electromagnetic oscillation – wave – passes through a mass of polar and charged molecules, such as those composing biological tissue induces a forced-oscillation on each of these particles that it meets and transfers to each of them a tiny part of its energy. This induced oscillation will be most intense on the free particles which carry a net electric charge such as the free (mobile) ions that exist in large concentrations in all types of cells or extracellular biological tissue determining practically all cellular/biological functions [1], [2]. The induced oscillation will be much weaker or even totally negligible on the polar biological macromolecules and the water molecules that do not have a net charge and additionally are usually bound chemically to other molecules.

After each such event of interaction between the wave and a charged or polar particle, the remaining wave continues on its way through the tissue possibly scattered by a tiny angle and reduced by a tiny amount in its amplitude/intensity. After large numbers of such events, depending on the tissue's mass, density, and the number of polar/charged molecules, the remaining wave, if any, leaves the tissue as a scattered wave of reduced amplitude/intensity.

When the amplitude/intensity *E* of the oscillating field or wave is decreasing after interaction with the charged/polar molecules of a medium, its energy density decreases as well, according to the equation for the energy density of a plane, harmonic electromagnetic wave (as those usually produced by “Thomson” circuits):
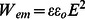
(1)
*W_em_* is the total energy per unit volume of the electromagnetic wave, and *E* the intensity of the electric component of the wave within a medium with relative permittivity *ε*. *ε_o_ = *8.854×10^−12^ C^2^/N⋅m^2^ is the vacuum permittivity.

That means that a part of its energy per unit volume is transferred to the charged/polar molecules of the medium.

The amount of energy absorbed by a single free ion within biological tissue will manifest itself as kinetic energy of the forced-oscillation induced on that particle. The maximum kinetic energy of the forced-oscillation is given by:



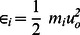
(2)where, *m_i_* is the ion mass which in the case of a Na^+^ ion, is *m_i_* ≅3.8×10^−26^ kg. *u_o_* is the particle's maximum velocity of the forced-oscillation assumed to be equal to ≅0.25 m/s,which is the drift velocity of Na^+^ ions along an open trans-membrane sodium channel, as calculated by patch-clamp ionic current measurements through open channels [3]–[5]. This maximum velocity (and kinetic energy) of the free ion is independent of the frequency of the external field [5], [6].

From Eq. (2) we get that the energy absorbed by a single ion due to the interaction with the electromagnetic wave, is: 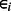 ≈1.2×10^−27^ J.

Considering that the concentration of free ions within cells is on the order of 1 ion per nm^3^ [1] and a typical cell volume up to 10^3^ μm^3^, a single cell contains about 10^12^ free ions and thus it will absorb about 10^12^×10^−27^ J = 10^−15^ J. A human body of average size consisting of ∼10^14^ cells, will absorb about 10^14^×10^−15^ = 10^−1^ J. For waves emitted by a supposed unidirectional antenna operating with 1 W ( = 1 J/sec) output power, (thereby transmitting energy 1 J per sec) it takes about 10 human bodies in sequence in order to be totally absorbed, according to the above mechanism, which seems a reasonable result.

But as mentioned already, except of the energy absorbed by mobile ions within biological tissue there will be additional energy absorption by the water dipoles and the charged or polar macromolecules like proteins, lipids, or nucleic acids, which will also be forced to oscillate by the applied field. While we can have an estimation as shown above for the energy absorbed by mobile ions, we are unable to estimate much smaller amounts of energy absorbed by charged or polar biological molecules. These smaller amounts of energy may be of decisive importance for the biological effect.

Even if we could accurately estimate macroscopically the amount of absorbed energy by a whole organ (e.g. by measuring an increase in temperature if any), again the biological effect depends basically on which specific bio-molecule(s) will absorb a certain amount of energy during a certain time-interval and this is impossible to discern. For example, when radiation is absorbed by lipids the damage will most likely be less than when the same amount of energy is absorbed within the same time-interval by enzymes and potentially even smaller than when absorbed by nucleic acids – especially DNA. Moreover, the situation becomes even more complicated in case that the biological effects are indirect. For example, a damage in the DNA may be due not to the energy absorbed directly by the DNA molecule but due to a conformational change in a membrane protein leading to irregular alteration of intracellular ionic concentrations [5], [6] and this in turn giving a signal for a cascade of intracellular events causing irregular release of free radicals or DNases which finally damage DNA (indirect effect).

Thus, even if we were able to determine the total amount of energy absorbed by an organ, tissue, or even a single cell during a certain time-interval, we still are not able to know the biological effect because this depends on the amounts absorbed by a variety of different biomolecules presenting widely varying interactive sensitivities to the radiation. In regard to ionizing radiation, this is well established. More specifically, it is well known that the biological effects of ionizing radiation depend a) on the type of ionizing radiation; it is known that equal doses (absorbed energy per unit mass of biological tissue, in Gy = J/kg) of different radiation types (e.g. alpha, beta, gamma, x, etc) absorbed during the same time-interval, result to different biological effects on the same type of biomolecule/tissue, b) on the type of biomolecule/tissue that absorbs a certain dose at a certain rate; a certain dose of a specific type of radiation – absorbed within a certain time-interval – will induce different effects on different biomolecules/tissue-types depending on their sensitivity and size [7], [8], [9]. We may then reasonably speculate that respectively, different types of non-ionizing radiation of the same *SAR* (differing between them in modulation, frequency, polarization, wave shape, etc) will induce different effects on a given type of biomolecule/tissue and moreover, that sensitivity of different biological molecules plays a crucial role in regard to the possibility of damage by a specific type of non-ionizing radiation at a certain *SAR* as well. These important issues are not addressed by *SAR* dosimetry.

Thereby, it follows that in the case of biological matter, the amount of absorbed energy as well as the rate of its absorption (*SAR*) does not determine the biological effect.

### The absorbed energy is normally well below the thermal level

While we are unable, as explained, to calculate accurately (microscopically) the absorbed energy at cellular level, we can estimate macroscopically with some satisfactory accuracy the energy absorbed by a whole body, or organ or tissue. But as we shall show (in the next section of the present study), only when the absorbed energy is large enough to cause measurable temperature increases. This naturally occurs when the absorbed radiation has a frequency above the lower limit of infrared which is about 3×10^11^ Hz [2]. Man-made microwave radiation used in modern telecommunications and other applications with frequencies 10^8^–10^10^ Hz cannot directly cause temperature increases in biological tissue unless it is of large enough power density (well above 1 mW/cm^2^). Radiation of even lower frequency would need to be of even larger power/intensity to produce thermal effects. Usual microwave intensities in modern human environment (mainly due to mobile telephony handsets and base station antennas, Wi-Fi, and radio-television station antennas) range between 0.01 μW/cm^2^ and 100 μW/cm^2^. Man-made radiation that has neither the frequency nor the intensity to cause thermal effects, it can still be absorbed – as explained above – in much smaller quantities by inducing forced-oscillations on polar molecules and free charges such as the free ions within all living cells. These forced-oscillations are superimposed on the thermal vibration of the same particles, increasing their thermal energy. But as we shall demonstrate, the energy of the oscillations induced by external EMFs at environmental exposure levels (intensities) is normally millions of times smaller than the average thermal energy *kT* of the molecules within a biological tissue, and thus does not produce measurable temperature increases. Although these induced oscillations (with kinetic energy usually millions of times lower than the average thermal energy) normally do not add to tissue temperature, they can still cause severe biological alterations (such as DNA damage) without heating the tissue [10]. These are called “non-thermal effects” and if not properly equilibrated by the organism's immune and other compensatory systems, they may very well result in health effects [11]–[14].

The maximum velocity of the ion's induced vibration is assumed to be, *u_o_* ≅0.25 m/s as explained, and the corresponding maximum kinetic energycalculated by Eq (2), is: 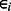 ≈10^−27^ J.

This ion possesses also an additional average velocity *u_kT_*, due to its thermal energy. The average kinetic energy of a single-atom molecule/free ion due to thermal motion [15], is:
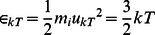
(3)


which gives: 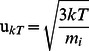
(4) where *T* = 310 ^o^K (the temperature of the human body 37^°^C), *k = *1.381×10^−23**^J⋅K^−1^ the Boltzmann's constant, and *m_i_* the ion's mass (*m_i_* ≅3.8×10^−26^ kg for Na^+^ ions) [5], [6].

From Eq (3), (4) we get:
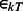
≅6.4×10^−21^ J, and*u_kT_* ≅0.58×10^3^ m/s.

Comparing the values of the above two different velocities/energies we find that, the maximum velocity acquired by a free ion within a cell due to an environmental EMF is normally about 2.3×10^3^ (≅ 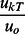) times smaller than its average thermal velocity and its corresponding maximum kinetic energy 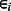 = 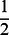 *m_i_ u_o_^2^* induced by the environmental EMF is about 5.3×10^6^ times smaller than the average thermal energy 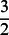 *kT* of such a particle. The average values of the environmental EMF-induced velocity and kinetic energy are even smaller than the above average thermal values.

Thereby, we have shown that oscillations induced on biological molecules by environmental EMFs do not usually contribute to the tissue temperature, except if these fields were millions of times more powerful, like for example the fields within a microwave oven operating at about 1000 W and focusing all of its radiating power within its cavity, in contrast to e.g. a GSM (Global System for Mobile telecommunications) mobile phone (∼0.1–1 W) or even a mobile telephony base station antenna (∼10–100 W) radiating (and distributing their energy) in all directions within wide angles.

Except of the tissue heating by high-power microwave radiation, the induction of small temperature increases on the order of 0.15–0.3°C has been reported after exposure of biological samples (*C. elegans*) to continuous wave 1 W, 1 GHz microwave radiation within a Transverse Electro-Magnetic (TEM) cell [16]. Nevertheless, in real exposure conditions as e.g. in the case of a GSM mobile phone during normal “talk” operation the average power density even in contact with the antenna hardly exceeds 0.2–0.3 mW/cm^2^ and does not induce temperature increases at a 0.05°C level as shown by use of a sensitive Hg thermometer with 0.05°C accuracy [17], [18]. Similar findings are also presented by other experimenters [19], [20]. Human exposure from base station antennas at a distance of a few meters is normally of even lower power densities.

Thus, environmental man-made EMFs are indeed unlikely to induce temperature increases in biological tissue, not even at the level of 0.05°C. Even the well-established thermal effect of “microwave hearing” attributed to thermo-elastic waves induced within the human/animal head by pulsed microwave radiation is calculated to correspond to temperature increases at a threshold of only 5×10^−6^°C [21]. Moreover, in the present paper it is shown theoretically that the energy absorbed by moving particles (free ions) within biological tissue due to environmental EMFs is millions of times smaller than the average thermal energy of such particles. Therefore if any temperature increases occur within biological tissue during exposure to environmentally accounted EMFs, they will normally be several orders of magnitude below 1°C and thus are not detectable.

The fact that the energy absorbed by living organisms due to the action of environmentally accounted man-made EMFs is indeed millions of times smaller than the average bio-molecular thermal energy, is the main reason why initially it was believed by scientists and authorities that environmental EMFs could not induce any biological effect [22]. That was based on the arbitrary hypothesis that an external EMF can only affect a living organism by increasing its temperature. Therefore, any non-thermal biological effect due to environmental man-made EMFs should be either not real, or attributed to hypothetical mechanisms such as the “stochastic resonance” by which biological matter can allegedly amplify small bits of information in a “sea” of white (thermal) noise by using the energy of the noise [23]. Such speculations – although they cannot be excluded – are not anymore necessary, since it is now known that due to forced-oscillation, the coherent motion (in the same direction) of several charged particles (free ions) within a cell in phase with a polarized external field can exert a larger resultant force on certain sensors (such as e.g. the voltage-sensors of electro-sensitive ion channels on cell membranes) than the mutually extinguishing forces on the same sensors due to their random thermal motions in all possible different directions [5], [6].

Even though some scientists still express skepticism regarding the existence of non-thermal effects [24], there is already a large and constantly increasing number of studies indicating that environmental man-made EMFs can produce severe biological alterations such as DNA damage without heating the biological tissue [10], [11], [17]–[20], [25]–[32]. This can take place through non-thermal mechanisms that involve direct changes in intracellular ionic concentrations or changes in enzymatic activity [5], [6], [33]–[35]. DNA damage may lead to cancer, neurodegenerative deceases, reproductive declines, or even heritable mutations. Brain tumors, decrease in reproductive capacity, or symptoms reported as “microwave syndrome” (headaches, memory loss, fatigue, etc), are observed among people exposed to mobile telephony radiation during recent years [30], [36]–[45]. Recently the International Agency for Research on Cancer (IARC) has classified RF/microwave EMFs as “possibly carcinogenic to humans” [46].

### The physics and biology of *SAR*

Usually, *SAR* values are reported in papers regarding exposure of biological material to RF EMFs, without any information about their calculation and without reporting the corresponding error.

As already mentioned, *SAR* is defined as the ratio of the absorbed power *P*, per unit mass of tissue, (in W/kg). To be more accurate, since electric power is not equally absorbed by different parts of biological matter, *SAR* is defined as the incremental power *dP* absorbed by an incremental mass of the tissue *dm* contained in a volume element *dV* of a given density *ρ* [47]:
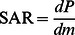
(5)where *dm*  =  *ρ dV*, (*ρ* in kg/m^3^).

Using Ohm's law:
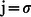
(6) where *j* is the electric current density (in A/m^2^) within the tissue due to the internal electric field *E* generated within the tissue, and *σ* the specific conductivity of the tissue (in S/m), relation (5) after operations (see Appendix S1), becomes:



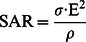
(7)From the derivation of the last relation for *SAR* (Appendix S1) it is obvious that the quantities: *j* (generated current density), *E* (generated internal electric field), *ρ* (tissue density), *σ* (tissue conductivity) are assumed to be constant within an organ (e.g. eye) or a group of organs (e.g. head) of a living body where we want to calculate *SAR*. This, of course, is an oversimplification since every organ or group of organs consists of many different types of biological tissue and all the above quantities vary significantly between different biological tissues and even within a single type of tissue and within a single cell.

More specifically, conductivity varies for different tissues and different field frequencies. For example at a frequency of 1 GHz, conductivity in different tissues of the human body can vary from about 0.04 S/m (bone marrow) to about 2.45 (cerebro-spinal fluid). Moreover the conductivity of a given tissue type increases considerably and non-linearly with frequency (up to a hundred times for a frequency range between 10^5^–10^10^ Hz) [48]. Even within a single cell, conductivity can have large variations from 10^−7^ S/m (cell membrane) to 0.5–1 S/m (cytoplasm, extracellular aqueous solution) [49], [50].

In addition, the available data on tissue conductivity are collected from measurements on dead animals and include large variations in relation to both tissue type and frequency range [48], [51]. These variations become even larger at *in vivo* conditions in alive animals. Higher conductivity values up to ∼300% than those previously reported, were recently measured in porcine organs of just sacrificed animals. The differences were attributed to the fact that the organs were still alive and filled with blood during the measurements in contrast to the previous studies which were performed on dead organs [52]. Moreover, the electrical properties of tissues – especially of the head – in all animals change with age. The relative permittivity of an adult human brain is calculated to be around 40 while the corresponding value for a young child's brain is between 60 and 80 resulting in almost double the radiation absorption and *SAR* [53], [54].

Moreover, human tissue density varies from about 900 kg/m^3^ (fat) to about1200 kg/m^3^ (tumor) between different soft tissue types and reaches a value of about 1800 kg/m^3^ for bones [51].

From this analysis it follows that Eq. (7) provides a poor definition of *SAR* due to the large variations of the related quantities, regardless of the estimating method. Thus, any estimating method for *SAR* based on Eq. (7) (see next section) includes a very large uncertainty.

For an homogeneous medium (thus neglecting again the local density variations) with specific heat *c*, [in J/(kg⋅K)] (thus neglecting also the local variations in the specific heat) and by use of a form of the calorimetry law:



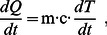
(8)


equation (5), becomes:
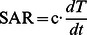
(9)where: 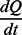 is the wave power, transformed into an incremental amount of heat *dQ*, within the tissue of mass *m*, producing an incremental temperature increase *dT* during the incremental time interval *dt*.

For a measurable time interval *δt* and a corresponding measurable temperature increase *δT*, Eq. (9) can be written as:
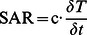
(10)


Since variations in specific heat within biological matter are usually much smaller than corresponding variations in conductivity [48], [51], [55] resulting in a much more uniform temperature than electric field distribution, Eqs. (9), (10) provide a better way for *SAR* estimation and, consequently, definition.

In addition, while differences in internal electric field intensity are retained during the whole exposure period since they depend on tissue permittivity which has large variations even within a single cell, differences in temperature between different locations of a tissue or organ are extinguished short time after the beginning of a constant exposure and temperature gets evenly distributed within a whole organ or even body. Moreover, while tissue conductivity and permittivity/internal electric field change significantly with different frequencies of the external field/radiation, specific heat is independent from the external field and depends only on tissue properties. In case of exposure to microwave radiation which includes more than one different frequencies (carrier, pulse, modulation frequencies), conductivity and internal field intensity depend on different simultaneous frequencies and their accurate estimation becomes, in any case, extremely complicated.

Even if we consider only one frequency and additionally neglect internal electric field intensity and density differences, conductivity variations alone result in a considerably larger variability of *SAR* as calculated by Eq (7) than by Eq. (10). For example, most organs/parts of the human/animal body contain both muscle and fat tissues. While at 1 GHz muscle conductivity (∼1.006 S/m) is about 1760% higher than fat conductivity (∼0.054 S/m), muscle specific heat (∼3.5 kJ/kg⋅K) is only 56% higher than fat specific heat (∼2.3 vkJ/kg⋅K). This would result to a ∼1700% larger variability in the *SAR* of this specific organ or part of the animal body when estimated by Eq (7) than when estimated by Eq. (10). At smaller frequencies conductivity variations increase considerably resulting in an even larger variability in the *SAR* calculation while specific heat has the same value. For example, at 10 MHz the above difference in *SAR* variability (∼1700%) between Eqs (7) and (10) becomes∼2125% (or 21.25 times larger variability in *SAR* value according to Eq (7) than according to Eq (10)) [56], [57]. If we add variations in internal electric field intensity and tissue density we may have hundreds of times larger variability in *SAR* values according to Eq (7) than according to Eq (10). Thus, while variation in *SAR* calculation according to Eq (10) is restricted to measurement errors and the assumption that *c* has the same value throughout the tissue, which somehow can be tolerated, corresponding variation in *SAR* according to Eq (7) includes similar errors plus tenths or even hundreds of times larger variability. This shows exactly that the only way to estimate *SAR* with some satisfactory accuracy is by measuring macroscopically the corresponding temperature increases – if any – within biological matter.

Therefore, it follows that *SAR* actually applies only to thermal effects and it actually expresses the rate by which electromagnetic energy from an external electromagnetic wave/field is converted into heat within biological matter. But as we have shown already, man-made electromagnetic fields at environmental levels do not normally cause thermal effects (measurable temperature increases within exposed biological matter) and this is in agreement both with experimental studies [10], [11], [17]–[20], [28], [31], [32], [58] and plausible proposed mechanisms for the action of EMFs on cells [5], [6], [33]–[35]. Thereby, it follows that, *SAR* is not a proper measure to describe the biological activity of man-made electromagnetic fields at environmental levels.

### The estimation of *SAR*

*SAR* is estimated by one of the following ways, [59]: 1) Insertion of micro-antennas or probes into the tissue, which detect the internal electric field. If the conductivity and the density of the tissue are known (assuming they have constant values) and neglecting local variations in internal field value, the *SAR* can be computed from Eq. (7). 2) Insertion of miniature thermal probes into the tissue. If a change *δΤ* in the temperature of the tissue is recorded, caused by the radiation/field during a time interval *δt*, and the tissue is supposedly homogeneous with known specific heat, then *SAR* can be computed by Eq. (10). 3) Numerical modeling, like the Finite Difference Time Domain, (FDTD) method, simulating the spatial distribution of the radiation energy within an object with the dimensions of the human body and computing SAR by Eq. (7). All the above ways/methods include significant error.

The first way does not take into account the local variations of conductivity, density and internal electric field within the tissue as explained already. Therefore this approach to *SAR* assessment is highly simplified compared to the complexity of real biological matter.

The second way provides a better approximation since temperature is much more evenly distributed within biological tissue than conductivity or electric field. But this assumes that there are detectable temperature increases (*δT*) – thus assuming solely thermal effects – while usually there are not as already shown, and additionally, the insertion of needles (thermal probes) disturbs any living tissue/organ and distorts its physical properties in unpredictable ways.

The third way, like the FDTD method, considered the best, simulates numerically the tissue by use of computers, dividing its volume into little pieces (voxels). Each voxel is assigned to certain values of conductivity, permittivity and density. Then *SAR* is again computed according to Eq. (7). Since within each voxel conductivity, permittivity, and density are assumed to be constant, this way also (alike the first way) represents an approximation and simplification. This is why earlier *SAR* estimations, defining the current criteria for whole body average *SAR* [60], are questioned by more recent and more accurate FDTD calculations [61]–[63]. In any case, all methods of simulation, no matter how much improved, are and will always be, highly simplified compared to living tissue, since they can never take into account the countless variations in the physical parameters of living matter especially at cellular level.

It follows that all the existing methods for *SAR* estimation, and especially those based on Eq (7), have serious deficiencies.

In addition, it becomes evident that all methods for *SAR* estimation are highly sophisticated, complicated, and time-consuming, so that *SAR* cannot be readily measured/calculated by use of the equipment of an ordinary radiation/biological laboratory.

### The non-linearity between electromagnetic exposure and biological effect

Dosimetry in science is necessary in order to find a quantitative relationship between cause and effect. The more well defined this relationship, the more useful the dosimetry. By knowing the relationship between cause and effect, we can predict the effect for different values of the magnitude of the cause for which we might have no experimental data. The most accurate prediction is when the cause-effect diagram is a straight line, e.g., where doubling the cause doubles the effect. In such a case we say that the cause-effect relationship is linear.

The biological/health effects from man-made EMFs/non-ionizing radiation, do not follow a linear dose-response (or cause-effect) relationship according to the experimental evidence. Experiments have shown that, the absorption of a larger amount of energy by the same mass of a given tissue and within the same time-interval, does not necessarily induce a larger biological effect. In other words, a more intense field or larger *SAR* does not necessarily relate to a larger biological response or consequent health effect.

The non-linearity of biological effects of man-made EMFs, and especially RF/microwave fields modulated by Extremely Low Frequency (ELF) signals (0–300 Hz), where the largest effects do not correspond to the largest *SAR* or intensity values,has been reported in several experiments since the mid-seventies [64]–[67]. Since then, it has been repeatedly verified by numerous studies [18], [31], [68]. For example, in one of the studies regarding effect of GSM radiation on the permeability of the blood-brain barrier in rats, and although other studies found no effect on the blood-brain barrier [69], it was reported that the strongest effect was produced by the lowest *SAR* values which corresponded to the weakest radiation intensity [68].

Moreover, in several studies, regions of increased bioactivity called “windows” were recorded, where the biological effects reach a maximum compared to the effects at smaller or larger values of a physical parameter like the intensity (and thus *SAR*) or frequency of the radiation. The “windows” represent an as yet unexplained phenomenon of the biological effects of EMFs, where increased bioactivity appears within certain values of a physical parameter of the field/radiation, but not for lower or higher values of this parameter [18], [31], [67], [70], [71]. Recently an intensity window on the biological effects of mobile telephony radiation was discovered where the effect on DNA damage was more intense around the value of 10 μW/cm^2^ in terms of the microwave – carrier – radiation intensity, than for intensities larger than 250 μW/cm^2^. More specifically, the borders of this “window” were found to be located between 8 and 28 μW/cm^2^ [18], [72].

In such a case of non-linearity, the inaccuracy between cause and predicted effect can be large. We should not make it even larger by using a dosimetric quantity that is further inaccurately estimated such as the *SAR*. Instead, we should at least use a measure that can be known more precisely.

Such a more precise quantity is the radiation/field intensity on the surface of the biological object as measured by any qualified and calibrated radiation/field meter (plus the additional physical parameters of the field/radiation which can also be accurately known, such as pulse and/or carrier frequency, waveform, modulation etc).

Any inaccuracy in the intensity measurement, as for example it may occur within an antenna's reactive near field, would be further increased in a corresponding *SAR* estimation. More specifically, if the electric field intensity *E* varies significantly within an antenna's near field, the corresponding *SAR* value depending on *E* ^2^ (according to Eq. 9) will include this variation plus the variation in the conductivity and density of the biological matter.

The reason for the non-linearity between electromagnetic exposure and biological effect may well be exactly the fact that the amount of absorbed energy or the rate of its absorption (*SAR*, field intensity) does not determine the biological effect as we explained. Indeed, the amount of absorbed energy during a certain time-interval (in other words the rate of energy absorption) increases with increasing intensity or *SAR*. If the corresponding biological effect increased proportionally, there would be no “windows” or other non-linear effects in regards to intensity or *SAR*. Nevertheless such effects exist and they are repeatedly recorded since the mid-seventies.

Finally, the non-linearity of several types of biological effects has been reported regardless of exposure to EMFs, and in response to a variety of external factors such as ionizing radiation, physiological, pharmacological, or chemical agents, environmental contaminants, etc [73]–[78], indicating that a non-linear response to environmental factors is intimately associated with living matter.

## Discussion and Conclusions

As explained, the only way that *SAR* can be calculated more accurately is through Eq. (10) by measuring temperature increases within the exposed biological tissues. But as shown in the present study, man-made EMFs at environmental levels do not normally cause measurable temperature increases except if they were millions of times more powerful. Thus, *SAR* – although not formally introduced specifically as a thermal term – actually refers only to thermal effects while the vast majority of the reported effects from environmental EMFs are non-thermal.

Moreover, as we explained, even if *SAR* for a whole body, organ, tissue, or even a single cell could be accurately estimated for exposures to environmentally accounted man-made EMFs, the biological effect depends on which specific biomolecule(s) absorb certain amount of energy within a certain time-interval, and this is impossible to discern.

Further, *SAR* offers no information at all with respect to frequency, waveform, or modulation of the EMF/radiation although these parameters are directly related in the literature to biological (and consequent health) effects. More specifically, it is repeatedly reported that amplitude-modulated or pulsed fields are more bioactive than non-modulated or continuous fields of the same carrier frequency, and the same average intensity (and thus the same *SAR*) [31], [64]–[67], [79]–[87]. Moreover, it is reported that signals of the same *SAR* but with different modulation types produce different effects in the same biological sample [79], [81], [84]. Real voice-modulated electromagnetic waves are considered to be more bioactive than simulated/periodically-modulated waves of similar other parameters and of the same *SAR* [10], [28], [31], [58]. In some cases it is also reported that microwave radiation modulated in amplitude by an ELF field, produced similar effects with the specific ELF field alone [84].

A plausible explanation for the reported increased bioactivity of the ELF components of a microwave field can be given by the “ion forced-oscillation theory” [5], [6], according to which the bioactivity of oscillating EMFs is inversely proportional to the frequency of the field and directly proportional to the amplitude of the forced-oscillation induced on free ions in the vicinity of cell membrane electrosensitive channels within the exposed biological tissue. Moreover, according to the same theory, pulsed fields are twice as much bioactive than the corresponding continuous wave (CW) fields with identical other parameters [5], [6] and this explains the results of several studies reporting that pulsed fields are more bioactive than the corresponding CW ones [80], [82], [85], [86].

A significant effect of carrier and modulation/pulse repetition frequency in microwave radiation is also indicated by several studies which have reported that fields of the same *SAR* but of different carrier or modulation frequencies produced different biological effects on the same biological sample [71], [88]–[90].

The above evidence regarding the importance of modulation and frequency of EMFs in regard to their biological activity is in total contradiction with any *SAR* approach, since it becomes evident that *SAR* alone – even if accurately estimated – is inadequate for predicting the biological effect, and the type of modulation as well as the frequency (modulation/pulse, carrier) have to be considered.

Thus, not only the biological effect depends upon undetermined tiny amounts of energy/power absorption by specific biomolecules exhibiting different sensitivities to the specific external field/radiation, but, moreover, it depends upon characteristics of the field/radiation, not taken into account in *SAR* calculation, such as modulation and frequency. Moreover, as explained, *SAR* estimation encounters significant error, especially in the case of environmentally accounted man-made EMFs.

In contrast to *SAR*, the characteristics of the external field, (intensity, frequency, etc.), can at the very least be measured much more accurately in any case. In case that the biological object is exposed within the reactive near field of an antenna where large variations of the intensity occur, *SAR* would be even more inaccurately estimated.

For taking into account possible field distortion by the exposed biological object due to possible resonance phenomena and localized regions of enhanced radiation absorption, although such phenomena are not expected to alter significantly the field, radiation/field intensity measurements must be carried out both in the presence and in absence of the biological object and in different locations corresponding to different parts of its surface. In case that the measured values in presence and in absence of the object are significantly different between them, both sets of measured values must be reported.

Certainly, because of the usually accounted non-linearity in the response of living matter to different external/environmental agents and especially to EMFs, neither *SAR*, neither radiation/field intensity are expected to be precise predictors of the induced biological effects. But at the very least, radiation/field intensity can be readily and more accurately measured than *SAR* can ever be estimated, especially for environmentally accounted man-made EMFs.

We conclude that *SAR* should not be considered as a proper dosimetric quantity to describe non-thermal effects which constitute the vast majority of the effects produced by man-made EMFs in our everyday environment. *SAR* should only be used complementarily to intensity measurements and the methods of its calculation along with the corresponding error should always be reported so that the reader can have information about the variability of the stated *SAR* values. For the same reason the radiation/field meter type and model used to measure the exposing field should always be reported in papers plus the variability (e.g. standard deviation) of the measured intensity values.

As increasing evidence is being accumulated for intense biological activity of man-made EMFs with consequent adverse effects on the human health and the natural environment, the need for fast and reliable measurement/dosimetry of such fields is becoming demanding. Thus, the measurement/dosimetry of EMFs should be easily performed by biological/radiation laboratories around the world by proper use of accurate field/radiation meters which are readily available in the market and easy to be used by qualified scientists/technicians, and not be based on complicated, time-consuming, and largely inaccurate methods of *SAR* estimation that cannot be readily performed.

## Supporting Information

Appendix S1 (DOC)
