# Correction: Evaluation of Specific Absorption Rate as a Dosimetric Quantity for Electromagnetic Fields Bioeffects

**DOI:** 10.1371/annotation/58c704d9-7cc4-4e4b-873b-214e6e2655ba

**Published:** 2013-11-12

**Authors:** Dimitris J. Panagopoulos, Olle Johansson, George L. Carlo

An error occurred in Equation 6, j = *σ*. The correct Equation should be j = *σ E*. 

