# Supplementary material for: Evaluation of Specific Absorption Rate as a Dosimetric Quantity for Electromagnetic Fields Bioeffects
Source: PLoS One. 2013 Jun 4;8(6):e62663. doi: 10.1371/journal.pone.0062663 (PMC3672148; doi:10.1371/journal.pone.0062663)
Supplement: Appendix S1 — (DOC) [file pone.0062663.s001.doc]

**Appendix 1**

Derivation of Eq. (7):

From equation (5):  *SAR* = $\frac{dP}{dm}$

and neglecting thermal losses, the absorbed electric power can be expressed as the power of a generated internal electric current within the tissue/organ, *dP = dΨ⋅ i*, where *dΨ*, is an incremental voltage generated within the tissue/organ by the external electromagnetic field/wave corresponding to the absorbed incremental power *dP*, and *i* the current intensity - across an area *S* vertical to the current - corresponding to the incremental voltage *dΨ*. Thereby, Eq. (5) becomes:

*SAR* = $\frac{d\Psi\cdot i}{dm}$ (11) By multiplying both the numerator and the denominator by the area *S*, we get

*SAR* = $\frac{d\Psi\cdot i\cdot S}{dm\cdot S}$, or

*SAR* = $\frac{d\Psi\cdot j\cdot S}{dm}$ (12) where *j* = $\frac{i}{S}$ is the corresponding current density generated within the tissue/organ.

Since *dΨ = -E⋅ d r*, where *E* the generated internal electric field and *dr* a charge displacement in the direction of the generated current *i*, we get (neglecting the minus sign):

*SAR* = $\frac{E\cdot dr\cdot j\cdot S}{dm}$ (13)

But *dr⋅S = dV*, is the volume corresponding to the area *S* and the charge displacement *dr*, containing the mass *dm*, and $\frac{dm}{dV}$ = *ρ* is the tissue/organ density assuming it has a constant value. Thereby, eq. (13) becomes:

*SAR* = $\frac{j\cdot E}{\rho}$ (14)

Finally, using the Ohm’s law: *j = σ Ε* , from Eq. (14) we get Eq. (7):

*SAR =* $\frac{\sigma\cdot E^{2}}{\rho}$

It is obvious that in the above operations, the quantities *i*, *j*, *S*, *E*, *ρ*, and *σ* are assumed to be constant within the biological tissue/organ.
